# Supplementary material for: Trichomes on female reproductive tract: rapid diversification and underlying gene regulatory network in Drosophila suzukii and its related species
Source: BMC Ecol Evol. 2022 Jul 28;22:93. doi: 10.1186/s12862-022-02046-1 (PMC9331688; doi:10.1186/s12862-022-02046-1)
Supplement: Supplementary file 1 — Additional file 1: Figure S1. Oviprovector scales from additional strains. (A–C) Oviprovector scales from dissected ovipositors in three additional strains, Hilo, TMUS09, and TMUS18, of D. suzukii. (D–F) Oviprovector scales from dissected ovipositors in three additional strains, M4, NAR02, and NAR07 of D. subpulchrella. Scale bar indicates 50 μm. Figure S2. Diversification of oviprovector scales in D. suzukii and its related species. . (A–C) Oviprovector scales from dissected ovipositors in three additional strains, Hilo, TMUS09, and TMUS18, of D. suzukii. (D–F) Oviprovector scales from dissected ovipositors in three additional strains, M4, NAR02, and NAR07 of D. subpulchrella. Scale bar indicates 50 μm. Figure S3. svb expression in the developing ovipositor. (A) Schematic image of the developing female terminalia. Abbreviations indicate analia (An) and ovipositor (Ovi). (B) in situ hybridization of svb at 44 h APF in D. suzukii. (C) in situ hybridization of svb at 44 h APF in D. subpulchrella. (D) in situ hybridization of svb at 48 h APF in D. suzukii. (E) in situ hybridization of svb at 48 h APF in D. subpulchrella. Scale bars indicate 100 μm. Figure S4. Oviprovector scales of mutants of trichome-related genes in D. melanogaster. (A) Wildtype Canton-S. (B–C) Mutant phenotype of trichome patterning genes, sha (B), m (C) and tyn (D). Scale bar indicates 20 μm. [file 12862_2022_2046_MOESM1_ESM.pdf]

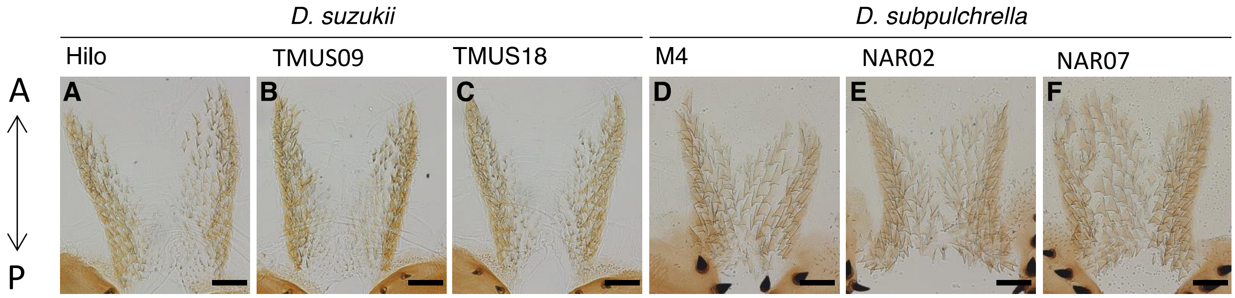


**Fig. S1 Oviprovector scales from additional strains.** (A–C) Oviprovector scales from dissected ovipositors in three additional strains, Hilo, TMUS09, and TMUS18, of *D. suzukii*. (D–F) Oviprovector scales from dissected ovipositors in three additional strains, M4, NAR02, and NAR07 of *D. subpulchrella*. Scale bar indicates 50 µm.

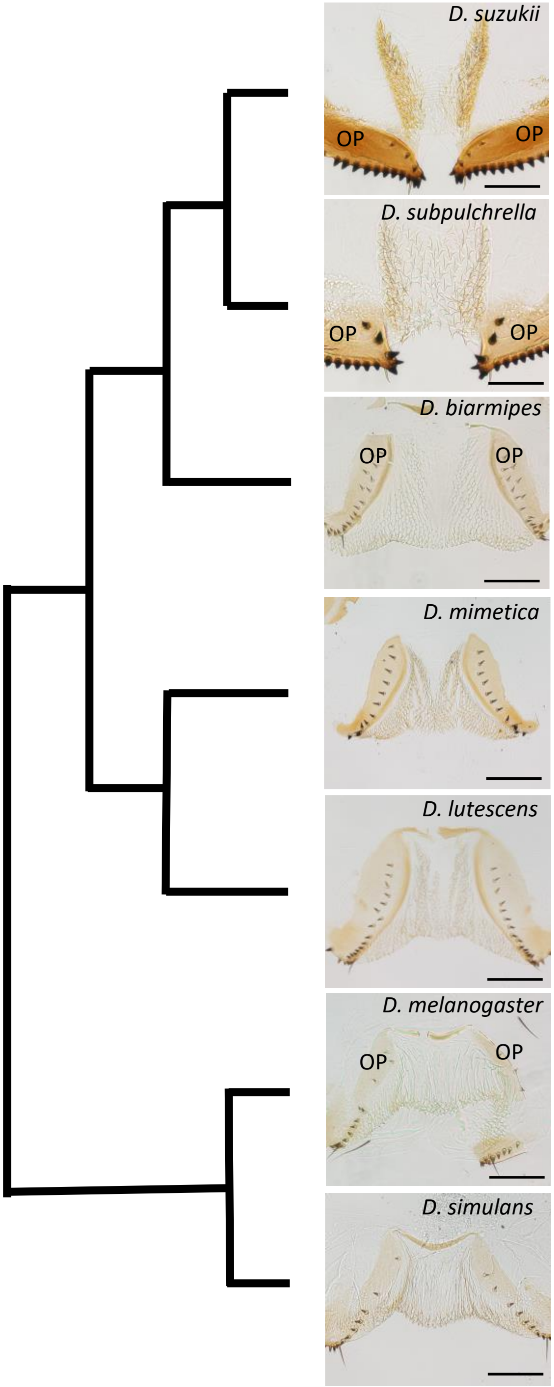


**Fig. S2** Oviprovector scales in *Drosophila suzukii*, *D. subpulchrella*, and five other related species. (A) Phylogenetic relationship and the images of oviprovector scales from dissected ovipositors. Tree topology is based on Suvorov et al. [57] and Finet et al. [58]. Scale bar indicates 100 μm.

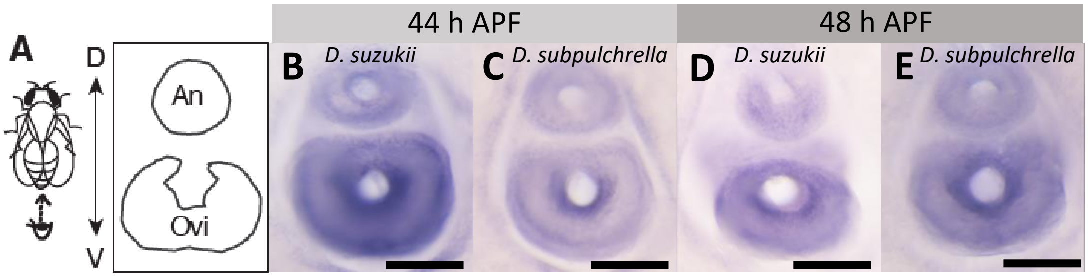


**Fig. S3** *svb* expression in the developing ovipositor. (A) Schematic image of the developing female terminalia. Abbreviations indicate analia (An) and ovipositor (Ovi). (B) in situ hybridization of *svb* at 44 h APF in *D. suzukii*. (C) in situ hybridization of *svb* at 44 h APF in *D. subpulchrella*. (D) in situ hybridization of *svb* at 48 h APF in *D. suzukii*. (E) in situ hybridization of *svb* at 48 h APF in *D. subpulchrella*. Scale bars indicate 100 μm.

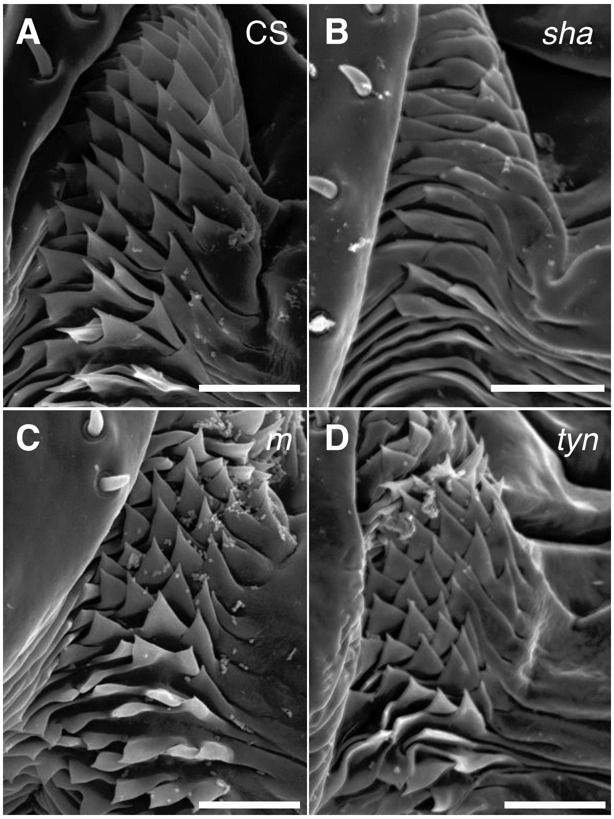


**Fig. S4 Oviprovector scales of mutants of trichome-related genes in *D. melanogaster*.** (A) Wildtype Canton-S. (B–C) Mutant phenotype of trichome patterning genes, *sha* (B), *m* (C) and *tyn* (D). Scale bar indicates 20 μm.
